# Deep learning algorithm based on analyzing the effect of posterior cervical vertebral canal decompression angioplasty in the treatment of ossification of posterior longitudinal ligament of cervical spine by CT image

**DOI:** 10.12669/pjms.37.6-WIT.4857

**Published:** 2021

**Authors:** Yang Liu, Jianjun Kong

**Affiliations:** 1 Yang Liu, Attending Physician. Department 9 of Orthopaedics, General Hospital of Jizhong Energy Xingtai Mining Group Co. Ltd., Xingtai, 054000, Hebei, China; 2 Jianjun Kong, Attending Physician. Department 9 of Orthopaedics, General Hospital of Jizhong Energy Xingtai Mining Group Co. Ltd., Xingtai, 054000, Hebei, China

**Keywords:** Cervical spine, Ossification of posterior longitudinal ligament, Hyperextension injury, Spinal stenosis rate, JOA improvement rate

## Abstract

**Objective:**

The paper uses the convolutional neural network algorithm in the deep learning algorithm to explore the therapeutic effect of surgical treatment of hyperextension injuries associated with ossification of the posterior longitudinal ligament of the cervical spine.

**Methods:**

In this retrospectively analyzed study 27 patients with hyperextension injury of the posterior longitudinal ligament of the cervical spine were selected from our hospital between August 2018 to July 2020. It included 21 males and 6 females; aged 36-79 years, with an average of 55.9 years.

**Results:**

Follow-up time of patients was 3-39 months, with an average of 17.4 months. The JOA score after surgery was significantly better than that before surgery (P<0.01), which was statistically significant; the improvement of JOA in patients undergoing anterior therapy was better than that in patients undergoing posterior therapy, which was statistically significant; the JOA improved in patients with minor violent injuries. The situation is significantly better than severe violent injuries, with statistical significance. The rate of postoperative JOA improvement was significantly correlated with the degree of nerve function retention of the injured spinal cord before surgery (P<0.01), and there was no significant correlation between the degree of spinal stenosis caused by ossification and the postoperative JOA improvement of patients.

**Conclusion:**

Convolutional neural network algorithm in the deep learning algorithm based on the cervical spine posterior longitudinal ligament ossification hyperextension injury was significantly improved after surgery. The less preoperative neurological damage, the postoperative neurological function, the degree of improvement, there was no significant correlation between the degree of spinal stenosis and the improvement of postoperative spinal cord function. For patients with ossification of the posterior longitudinal ligament, if there are neurological symptoms, early surgical treatment is recommended to relieve the compression, so as to prevent irreversible neurological damage caused by trauma.

## INTRODUCTION

Imaging examination can be used for posterior cervical decompression angioplasty. CT imaging can distinguish soft tissue and clearly show the location and size of epidural hematoma and the degree of bone marrow compression.^1^,^2^ Compression of the spinal cord for a long time will lead to permanent spinal cord injury and eventually paralysis. CT imaging can detect epidural hematoma as soon as possible.^3^ The introduction of CNN in CT imaging can clearly extract image features, segment target lesions, and quickly diagnose and treat diseases.^4^

In order to explore the therapeutic effect of surgical treatment of hyperextension injuries of the posterior longitudinal ligament ossification of the cervical spine, we selected patients with cervical hyperextension injuries with ossification of the posterior longitudinal ligament. We wanted to study its. clinical characteristics and postoperative improvement.

## METHODS

### General information

The paper retrospectively analyzed patients with cervical hyperextension injury who were treated with ossification of the posterior longitudinal ligament of the cervical spine from August 2018 to July 2020 in our hospital after IRB approval; date March 17, 2021 . This group of 27 patients, 21 males and six females; aged 36-79 years, with an average age of of 55.9 ± 9.6 years. As regards causes of injury, : two cases of injury from falling down, 7 cases of trauma from car accident, and 18 cases of accidental fall. Injury time: two hours to three days, average 27 hours. One case was accompanied by respiratory limitation on admission.

The imaging data of the patients were analysed and studied by using the hospital PACS system. OPLL type: continuous type 11 cases, segmented type eight cases, isolated type five cases, mixed type 3 cases; ossification segment: distributed in one segment eight cases, distributed in two segments five cases, distributed in three There were five cases in each segment, three cases in four segments, and five cases in five segments; MRI showed 16 cases of high signal in the spinal cord. Cervical spine CT showed 8 cases of “double shadow sign” in ossification.

Anterior cervical surgery was performed in eight cases, two cases underwent anterior cervical discectomy and decompression, bone graft fusion and internal fixation, and six cases underwent anterior cervical vertebral body subtotal resection; It was found that the posterior longitudinal ligament that was difficult to separate due to severe ossification was decompressed by the anterior floating method, and then the appropriate size of Cage or titanium mesh filled with autogenous bone fragments was implanted between the vertebral bodies, and an appropriate steel plate was selected to be placed in front for compression and fixation. Fifteen cases of posterior cervical surgery and 12 cases underwent posterior cervical spine decompression and plastic surgery; two cases had posterior cervical lamina decompression lateral mass screw internal fixation, and two cases underwent posterior cervical lamina reduction Lateral mass screw internal fixation + anterior cervical disc removal and decompression bone graft fusion internal fixation.

The neurological function of the patients was selected by the Japan Orthopaedic Association (JOA) score, and the neurological function of patients in the group before and after surgery was evaluated.

### Statistical analysis

SPSS 20.0 statistical software was used for analysis and comparison. The paired sample t test was performed on the improvement of JOA before and after surgery. The independent sample t test was performed on the JOA improvement of different surgical approaches and injuries. P <0.05 was considered statistically significant. Bivariate correlation analysis was used to compare the relationship between the degree of spinal stenosis and the rate of neurological improvement.

The accurate posterior cervical spinal canal CT image segmentation can greatly speed up the process of posterior cervical spinal canal segmentation, improve the efficiency of posterior cervical spinal canal diagnosis, and can rely at least on the experience of doctors, thereby reducing the probability of misdiagnosis.

The paper proposes a new fully convolutional neural network framework to segment the CT images of the posterior cervical spinal canal. This method can automatically learn important information for automatic segmentation from the training data, and this segmentation model can be a direct model from input original image to output image. Using the structural characteristics of the layered neural network, the architecture can automatically re-connect the learned features. The parameters of the model are greatly reduced compared to the convolutional neural network model in Chapter 3, while the model’s Image segmentation speed has been greatly improved. Experimental results show that this method can not only simplify the process of segmentation of the posterior cervical spinal canal CT image, but also improve the accuracy of segmentation. This operation process can be explained by mathematical formulas. Assuming that *X_ij_* is a data vector at position (*i,j*) in a certain layer, and *Y_ij_* is a data vector at a position in the next layer, then the relationship between these two data vectors is:


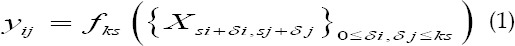


This function can be implemented in the form of interleaving different layers, and has the core size and step size to obey the rules:


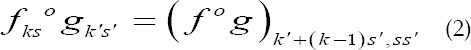


Next, define the loss function of the end-to-end convolutional neural network. The loss function defined in the paper is the sum of losses in the spatial dimension of the final layer, in the form:


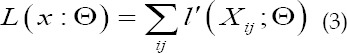


The method used in the paper is interpolation. When fused with the output information of the lower layer, deconvolution appears to be very efficient.

In a sense, the up sampling of the sampling factor f is the same as a convolution with an input stride factor of fraction 1/f. Therefore, as long as f is an integer, the natural way of up sampling is a reverse convolution with an output stride off, also known as deconvolution. In this way, the up sampling can be combined into an end-to-end network, so that each pixel of the image can be predicted, and the structure of the network is simplified.

The gradient will be the total gradient of each of its spatial components. Therefore, the random gradient descent loss calculated for the entire image will be related to the random gradient descent loss in each dimension. These receptive fields significantly overlap, and the loss is calculated for the output of these convolutions. Both forward data calculation and reverse gradient propagation are calculated layer by layer over the entire image, which is more efficient than independent block by block calculation.

The main idea of the active contour model is to use the energy function to parameterize the image to be segmented and initialize it on the segmented image. Under the action of the image force and the internal force of the control curve, the curve finally converges to the contour of the image. The mathematical expression of the energy function is:


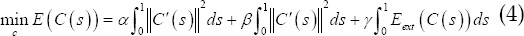


Here *α,β,γ* positive weighting factor adjusts the ratio of these three terms. For this energy functional, applying Eider LaGrange equation, and then introducing time variable, we can get the evolution equation of curve *C(s,t)*:


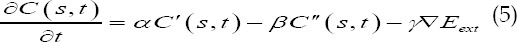


## RESULTS

**Fig.1:**
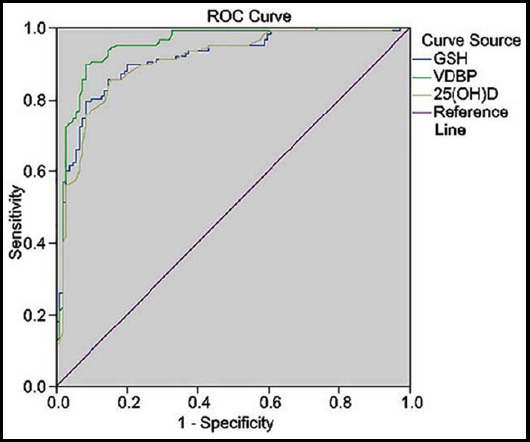
Linear regression analysis of the relationship between postoperative JOA improvement rate and preoperative JOA score.

The follow-up time of patients was 3-39 months, with an average of 17.4 months. The thickness of the posterior longitudinal ligament ossification is 2.5-11.93mm, with an average of 5.81±2.42mm; the stenosis rate of spinal canal loss was 22.8-87.5%, with an average of 53.24±18.09%. All the operations were successfully completed, and two cases of dyspnea occurred after the operation, and all improved with symptomatic treatment. One case of C5 nerve root paralysis occurred after posterior cervical spine double canal vertebral canal reconstruction, and recovered after three months of rehabilitation exercise. One case of cerebrospinal fluid leakage occurred after posterior cervical vertebral double-ported spinal canal reconstruction. Symptomatic treatment was performed after compression dressing and fluid replacement. The extubating was performed 11 days after the operation, and no postoperative infection occurred. The preoperative JOA score was 8.3±4.2 points, and the postoperative JOA score was 11.2±4.2 points. The postoperative JOA score was significantly better than that before operation (*P*<0.01), which was statistically significant; the JOA improvement in patients undergoing anterior surgery (53.51±13.04%) is better than the posterior (35.01±18.28%), the difference is statistically significant (Table-I); JOA improvement rate (49.22±15.83%) in patients with mild violent injuries is significantly better than patients with severe violent injuries (23.73±14.19%) (Table-II), the difference is statistically significant. The postoperative JOA improvement rate has a significant correlation with the degree of neurological function retention of the injured spinal cord before surgery (P<0.01) (Table-III and Fig.1), and the degree of spinal stenosis caused by the ossification of the posterior longitudinal ligament and the postoperative JOA There was no obvious correlation between the improvement rates (Table-III).

**Table-I:** Comparison of basic data and surgical efficacy of patients with different surgical approaches.

	*Front approach*	*Back approach*	*Front approach*
Number of patients (cases)	8	15	4
Gender: (Male: Female)	7:01	11:04	3:01
Age	54.9±10.4	57.7±9.8	51.3±6.9
Bone thickness	3.86±0.83	6.60±2.06	6.75±3.86
*Bone type*			
Segmented	4	3	1
Continuous	1	8	2
Isolated	2	2	1
Hybrid	1	2	0
*JOA score*			
Preoperative	11.38±2.33	7.07±4.37	6.50±3.70
Postoperative	14.25±1.83	10.00±4.28	9.75±4.92
JOA improvement rate	53.51±13.04%	35.01±18.28%	36.60±26.03%
*complication*			
C5 nerve root paralysis	0	1	0
Cerebrospinal fluid leakage	0	1	0

**Table-II:** Comparison of surgical efficacy of patients with different injury factors.

	*Slight violence*	*Number of severe violence patients (cases)*
Number of patients (cases)	18	9
Bone thickness (mm)	5.56±2.50	6.33±2.29
Spinal stenosis rate	52.29±17.57%	55.13±20.04%
JOA improvement rate	49.22±15.83%	23.73±14.19%

**Table-III:** Comparison of preoperative JOA score and postoperative JOA improvement rate.

Numbering	1	2	3	4	5	6	7
Preoperative JOA	1	2	3	3	3	3	4
JOA improvement rate%	25	33.3	7.1	28.6	14.3	14.3	15.4
Spinal stenosis rate%	69.9	37.9	87.2	72.2	65.2	28.7	78.1
Numbering	16	17	18	19	20	21	22
Preoperative JOA	11	11	11	11	12	12	12
JOA improvement rate%	66.7	66.7	50	50	40	60	60
Spinal stenosis rate%	44.1	62.4	62.5	41.6	48	61.1	43.7
Numbering	8	9	10	11	12	13	14
Preoperative JOA	4	4	6	6	8	11	11
JOA improvement rate%	38.5	7.7	27.3	36.4	66.7	33.3	50
Spinal stenosis rate%	87.5	50	60	22.8	46.7	53.4	65
Numbering	23	24	25	26	27		
Preoperative JOA	12	12	13	13	13		
JOA improvement rate%	60	40	50	50	75		
Spinal stenosis rate%	39.1	36.6	26.2	31.7	42.1		

## DISCUSSION

OPLL often causes compression of the spinal cord and nerve roots, causing neurological dysfunction. Compared with anterior surgery, posterior cervical decompression angioplasty has the characteristics of short operative time and flexible and simple operation. Posterior surgery can preserve the bilateral facet joints, and enable the posterior longitudinal ligament and the joint capsule ligament to bear a great load, conducive to the recovery of nerve function.^5^ Lee et al.^6^ found that, laminar decompression and fusion was significantly associated with the high average annual growth rate of OPLL. After spinal canaloplasty and laminar decompression and fusion, cervical lordosis was alleviated, which may be related to the strong internal fixation. In this study, after posterior cervical decompression angioplasty, nerve injury was reduced and postoperative nerve function was improved.

CNNs are widely used in image segmentation, image classification, and target image positioning in the medicine field. Some researchers have proposed that, combining pixel information of different scales can extract the best size information; some researchers believe that, reducing the size of the convolution kernel can improve the running speed of the neural network.^7^-^10^ The CT image reconstruction algorithm can effectively remove image noise and improve image quality, assisting physicians in diagnosing diseases. With the rapid development of deep learning, many deep learning networks are used to process CT images. Chen et al.^11^ used deep learning to process CT images, and the quality of image was improved. Xing et al.^12^ analyzed the role of deep learning in detection, segmentation, and classification of microscope images, and found that, deep learning had great potential. In this study, CNN was used to process CT images of patients undergoing cervical posterior decompression angioplasty, and deconvolution was used to predict each pixel of the image to simplify the network structure and reduce the loss in image calculation. Then, the energy parameters of the active contour model were used to parameterize the image. After the CT images were optimized, it was noted that, the JOA improvement of patients with anterior surgery (53.51±13.04%) was better than that of patients with posterior surgery (35.01±18.28%), and the difference was statistically significant (*P*<0.05). Oshima et al.^13^ analyzed the JOA scores of patients with cervical spondylotic myelopathy, and found that the preoperative JOA and pro-JOA scores were 10.8 and 10.6, respectively. After posterior decompression, the patients’ JOA and pro-JOA scores were 13.3 and 12.9, respectively. It was basically consistent with the results of this study that, the improvement rate of JOA after posterior decompression was increased, and the patient’s neurological function was effectively restored.

## CONCLUSIONS

For patients with ossification of the posterior longitudinal ligament, if there are neurological symptoms, early surgical treatment is recommended to relieve the compression, so as to prevent irreversible neurological damage caused by trauma. There was no significant correlation between the degree of spinal stenosis and the improvement of spinal cord function after operation. Nerve function was significantly improved after hyperextension injury of the posterior longitudinal ligament ossification of the cervical spine. The lighter the degree of nerve function damage before operation, the better was the degree of improvement of nerve function after operation.

### Author`s Contribution

**YL** conceived the study, literature review, data analysis, helped to draft the manuscript.

**JK** helped in design, data collection, article drafting & critical revision and is accountable for all aspects of the work in ensuring that questions related to the accuracy or integrity of any part of the work are appropriately investigated and resolved.
